# Labor Operation

**Published:** 1876-03

**Authors:** Rufus Linthicum

**Affiliations:** Calhoun, Ky.


*Professor E. S. Gaillard:*

Dear Sir,—On the 1st of July, 1872, I was called in consultation with my brother, Dr. O. M. Linthicum, to see a colored woman, aged about thirty years, living near Sebree City, Webster county, Kentucky. Near a fortnight before, she had been in throes of labor. She thought she had gone to full term of gestation, and sent for an old negress in the neighborhood. Afterwards she had two or three hard pains, then labor ceased. She felt exhausted, restless and uneasy, and gradually became anæmic. The child had passed out into the cavity of the abdomen instead of being expelled naturally. There was considerable prominence between the umbilicus and pubic arch, over which two points of ulceration had already set up, in the abdominal wall, through which hair had begun to protrude. The woman was in indigent circumstances, and we encouraged the ladies of the neighborhood to send her good and nutritious diet, and we provided her with wine and brandy, and decided to operate. On the evening of the 13th we cleared out her bowels with a brisk dose of epsom salts and ordered her to take no food on the following morning. We met early on the morning of the 14th, gave her a full brandy toddy, emptied her bladder by means of the catheter, put her upon the table, my brother administering the chloroform. I made an incision commencing a little below and to the left of the umbilicus, so as to carry my knife through the fibres of the rectus muscle, carried it down to within half an inch of the pubic bone, and took from the cavity a dead and partially decomposed fœtus, and a portion of the secundines, the most of which had been broken down by suppuration, a great deal passing through the opening at the time. From all appearances the fœtus was a full grown child at full term. She rallied well from the chloroform. We dressed the wound with interrupted suture and adhesive strips, and put her upon opium and fluids for seven days, at the expiration of which time I met my brother at her house, and we moved her bowels by clyster. She made a rapid recovery, not having a bad symptom from the time we operated. The rent has entirely healed, and she does not now require any abdominal support, and does all kinds of housework and field labor. Respectfully,

Rufus Linthicum, M.D.

